# Mis-Expression of a Cranial Neural Crest Cell-Specific Gene Program in Cardiac Neural Crest Cells Modulates HAND Factor Expression, Causing Cardiac Outflow Tract Phenotypes

**DOI:** 10.3390/jcdd7020013

**Published:** 2020-04-20

**Authors:** Joshua W. Vincentz, David E. Clouthier, Anthony B. Firulli

**Affiliations:** 1 Herman B Wells Center for Pediatric Research, Departments of Pediatrics, Anatomy and Medical and Molecular Genetics, Indiana Medical School, Indianapolis, IN 46202, USA; 2 Department of Craniofacial Biology, University of Colorado Anschutz Medical Campus, Aurora, CO 80045, USA; david.clouthier@cuanschutz.edu

**Keywords:** HAND1, DLX5, BMPs, transcriptional regulation, neural crest, craniofacial defects, cardiac defects

## Abstract

Congenital heart defects (CHDs) occur with such a frequency that they constitute a significant cause of morbidity and mortality in both children and adults. A significant portion of CHDs can be attributed to aberrant development of the cardiac outflow tract (OFT), and of one of its cellular progenitors known as the cardiac neural crest cells (NCCs). The gene regulatory networks that identify cardiac NCCs as a distinct NCC population are not completely understood. Heart and neural crest derivatives (HAND) bHLH transcription factors play essential roles in NCC morphogenesis. The *Hand1^PA/OFT^* enhancer is dependent upon bone morphogenic protein (BMP) signaling in both cranial and cardiac NCCs. The *Hand1^PA/OFT^* enhancer is directly repressed by the endothelin-induced transcription factors DLX5 and DLX6 in cranial but not cardiac NCCs. This transcriptional distinction offers the unique opportunity to interrogate NCC specification, and to understand why, despite similarities, cranial NCC fate determination is so diverse. We generated a conditionally active transgene that can ectopically express DLX5 within the developing mouse embryo in a Cre-recombinase-dependent manner. Ectopic DLX5 expression represses cranial NCC *Hand1^PA/^*^OFT^-*lacZ* reporter expression more effectively than cardiac NCC reporter expression. Ectopic DLX5 expression induces broad domains of NCC cell death within the cranial pharyngeal arches, but minimal cell death in cardiac NCC populations. This study shows that transcription control of NCC gene regulatory programs is influenced by their initial specification at the dorsal neural tube.

## 1. Introduction

Congenital heart defects (CHDs) afflict roughly 1% of newborns and ultimately affect the quality of life of more than 1 million adults in the United States [1]. Many CHDs affect the cardiac outflow tract (OFT) [2]. A significant portion of CHDs can, therefore, be attributed to developmental dysfunction of one of the main developmental progenitors of the OFT, known as the neural crest cells (NCCs). NCCs migrate from the dorsal neural tube throughout the developing embryo [2]. Different NCC subpopulations differentiate into distinct tissue types. The cardiac NCCs differentiate into smooth muscle and connective tissue to form portions of the aorta, pulmonary artery, and nascent ventricular septum. Although NCC have been well studied, the gene regulatory networks that drive NCCs cells to delaminate from the neural tube, migrate to their destinations, and differentiate into their specified cell type identity are still not completely understood.

The bHLH transcription factors HAND1 and HAND2 are expressed in both cranial and cardiac NCCs and loss of function studies establish them as critical players in craniofacial and OFT development [3,4,5,6]. *Hand1* NCC expression is observed post-NCC migration and resides within the ventral most cap of the pharyngeal arches that contribute to craniofacial structures as well as components of the OFT [7,8,9]. *Hand2* expression is also observed post-NCC migration. However, *Hand2* expression projects more dorsally and demarcates the entire ventral domain [6,8]. *Hand1* NCC expression directly depends on HAND2 and BMP-signaling mediated transcriptional activity [8,10,11]. *Hand2* NCC expression is dependent on the Endothelin-1 induced transcription factors DLX5 and DLX6, whose expression, with the exception of the *Hand1* expression-marked ventral cap domain, overlaps with that of *Hand2* [8]. HAND2 exhibits negative feedback upon *Dlx5* and *Dlx6*, excluding *Dlx5/6* expression from the ventral cap [6]. When *Hand2* expression is deleted from NCCs, *Dlx5/6* expression expands into the ventral cap, where it actively represses *Hand1* expression [8]. Collectively, these findings are consistent with a complex regulatory mechanism that allows for sub specialization of NCC within the pharyngeal arches critical for craniofacial and OFT morphogenesis.

Single-cell NCC analyses have proposed a cardiac NCC differentiation cascade in which *Dlx6* is transiently upregulated, followed by activation of *Msx2* and *Hand2*, and then activation of downstream cardiac markers including *Hand1* and *Gata6* [12]. However, this gene expression profile describes both the cardiac NCCs and the distal cap cranial NCCs. In this study, we utilize a novel conditional gain-of-function *Dlx5* allele to ectopically and persistently express DLX5 specifically within cranial NCCs. Using the NCC Cre driver, *Wnt1-Cre*, we demonstrate that, in cranial NCC populations, *Hand1* expression is repressed, whereas cardiac NCC *Hand1* expression is not directly affected. Significant amounts of cell death are observed in cranial NCC populations. However, cardiac NCCs exhibit less extensive apoptosis. This work advances our understanding of the unique transcriptional pathways at work within NCCs contributing to cranial and OFT tissues where gene expression within all NCC is interpreted differentially within specified NCC populations.

## 2. Materials and Methods

### 2.1. Transgenic Mice

The Indiana University Transgenic and Knock-Out Mouse Core generated the *CAG-CAT-FLAGDlx5* transgenic mouse line (denoted henceforth as *CAG-CAT-Dlx5)* on a C3HeB/FeJ background. Genotyping for this allele is performed via PCR using the forward primer Dlx5(F) 5′-CGGGACGCTTTATTAGATGG-3′ and the reverse primer Dlx5(R) 5′-TTGCATTGTTGGATTTCTGG-3′, which produces a 465 bp control band and the reverse primer SV40pA(R) 5′-CCCCCTGAACCTGAAACATA-3′, which produces a 295 bp amplicon that detects the presence of the transgene. After a 5 min incubation at 95 °C, the PCR conditions run are 95 °C 30 s, 55 °C 60 s, and 72 °C 60 s for 36 cycles. Use and genotyping of *Wnt1-Cre* and Gt*(ROSA)26Sor^tm1Sor^* alleles are previously reported [13,14]. Embryos were not selected for sex and were evaluated blindly for all analyses. Mice and other reagents are available from the authors upon request.

### 2.2. Cloning

The generated Cre-activatable transgene *CAG-CAT-Dlx5* was constructed by replacing the Myc-Twist1 cDNA of CAG-CAT-Twist construct [15] with the murine FLAGDlx5 cDNA.

### 2.3. Bone and Cartilage Staining, X-Gal Staining and Histology

Bone and cartilage staining was performed using Alizarin Red and Alcian Blue as previously described [6]. X-gal staining was performed as previously described [9,16,17,18].

### 2.4. Lysotracker and TUNEL

Cell death analysis on control and mutant embryos was performed as described [18,19]. *Lysotracker* (Life Technologies) was incubated with embryos as per the manufacturer’s instructions. Embryos were imaged in a well slide on a Leica DM5000 B compound florescent microscope. TUNEL analyses were performed upon sectioned embryos using the *ApopTag Plus* Fluorescein in situ Apoptosis detection kit (S7111 Chemicon International) as per the manufacturer’s instructions.

### 2.5. Immunohistochemistry

Immunohistochemistry was performed as previously described [17] using an antibody against TUBULIN β3 (β-TUBB3, Abcam). Images were collected on a Leica DM5000 B microscope and Leica Application Suite software.

### 2.6. In Situ Hybridization

Section in situ hybridizations were performed on 10-µm paraffin sections as described [20,21]. Antisense digoxygenin-labeled riboprobes were synthesized using T7, T3, or SP6 polymerases (Promega) and DIG-Labeling Mix (Roche) using the following plasmid templates: *Dlx5, Dlx6, Hand2*, *Sox9* (provided by Benoit De Crombrugghe), and *Ret* (provided by Jean-Francois Brunet).

### 2.7. Quantitative RT-PCR

Total RNA was isolated from E11.5 mandibular pharyngeal arches using the High Pure RNA Isolation Kit (Roche). This RNA was then used to synthesize cDNA using the High-Capacity cDNA Reverse Transcription Kit (Applied Biosystems). For qRT-PCR, cDNA was amplified using TaqMan Probe-Based Gene Expression Assays (Applied Biosystems) and the QuantStudio 3 Real-Time PCR System (ThermoFisher). Normalization to Glyceraldehyde 3-phosphate dehydrogenase (GAPDH)was used to determine relative gene expression and statistical analysis automatically applied by the instrumental software. Significance of qRT-PCR results were determined by a two-tailed students t-test followed by post hoc Benjamini-Hochberg FDR correction as automatically calculated by the QuantStudio 3 qRT-PCR thermal cycler software analysis package.

## 3. Results

### 3.1. NCC Expression of CAG-CAT-Dlx5 Results in Midface Clefting

**Figure 1:**
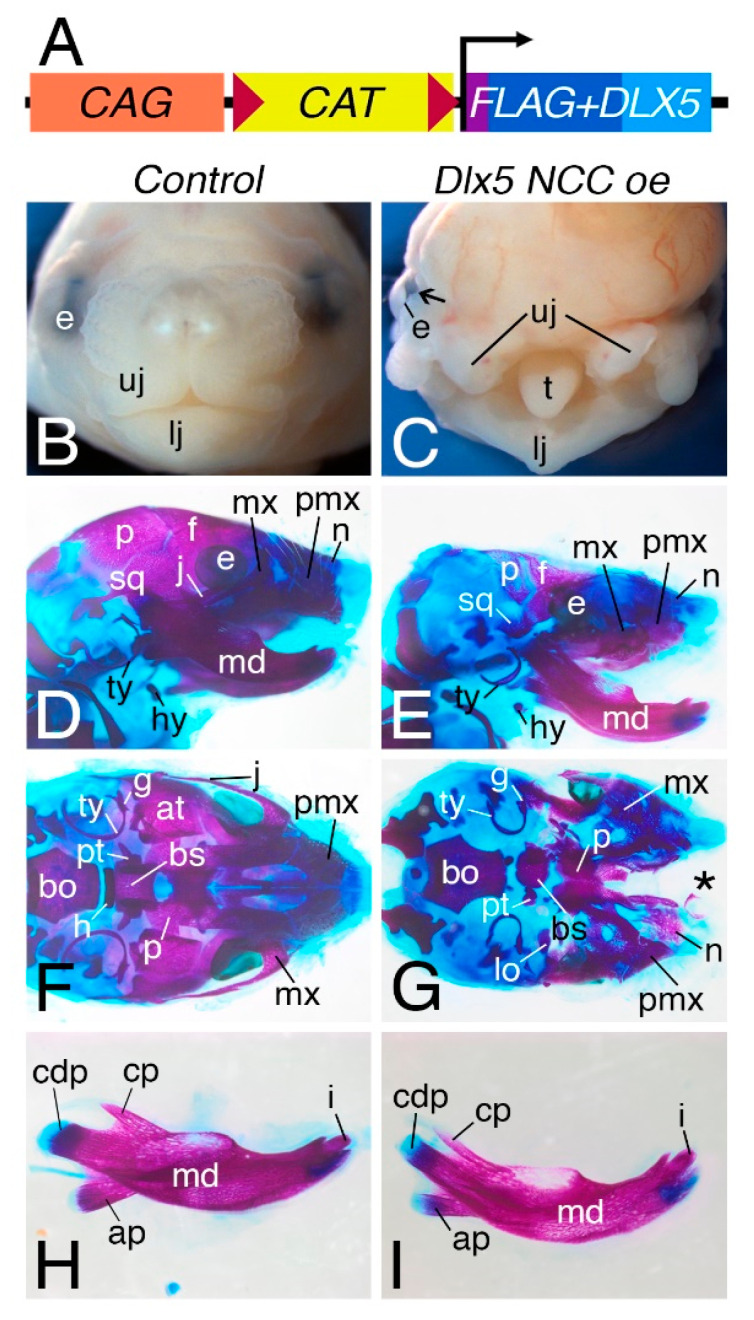
Neural crest cell (NCC)-specific *Dlx5* overexpression results in midface clefting. (**A**) Diagram of the *CAG-CAT-Dlx5* transgene. The *CAG* promoter drives expression of the *Chloramphenicol acetyl transferase* (*CAT*) gene that is flanked by loxP elements (triangles). Removal of Chloramphenicol Acetal transferase (CAT) via Cre-recombinase allows for persistent expression of *Dlx5*. (**B,C**). Anterior and ventral whole mount views, respectively, of E16.5 heads from Control (*CAG-CAT-Dlx5*) (**B**) and *Wnt1-Cre; CAG-CAT-Dlx5* (*Dlx5 NCC oe*) (**C**) embryos. Compared to control embryos, the midface of *Dlx5 NCC oe* embryos contain a large cleft separating the upper jaw (uj) into left and right sides and exposing the tongue (t). The lower jaw (lj) also appears misshapen and a coloboma (arrow) is present. (**D–I**). E18.5 control (**B**,**D**,**F**,**H**) and *Dlx5 NCC oe* (**C**,**E**,**G**,**I**) embryos stained with Alizarin Red and Alcian Blue to visualize bone and cartilage, respectively. (**D, E**) Lateral views of skulls. In control embryos, the parietal (p) and frontal (f) are observed, which abut the squamosal (sq) bone (**D**). In the jaw region, the nasal (n), premaxilla (pmx), and maxilla (m) bones are observed, with the zygomatic process of the maxilla abutting the jugal (j) bone of the zygomatic arch. The tympanic ring (ty) bone is also observed. In *Dlx5 NCC oe* mice, the parietal, frontal bones are hypoplastic, as is the tympanic ring and squamosal bones (**E**). The premaxilla, maxilla, and jugal bones are dysmorphic, though the mandible appears relatively normal. (**F**,**G**) Ventral view of skulls with the mandible removed. In contrast to control embryos (**F**), most skull base bones in *Dlx5 NCC oe* embryos are hypoplastic, including the basisphenoid (bs), pterygoids (pt), and alisphenoids (al). The palatine (p) bones fuse along the midline but are also hypoplastic, as are the tympanic rings (**G**). The dysmorphology of the maxilla along the midline is also apparent, along with the large midline cleft that now exists (*). (**H,I**) Lateral (intra-oral) view of the left mandible (md). The coronoid (cp), condylar (cdp), and angular (ap) processes of the mandible are present in both control (**H**) and *Dlx5 NCC oe* (**I**) embryos, as are the incisors (i). bo, basisoccipital bone; e, eye; hy, hyoid bone.

To test the hypothesis that mis-expression of a *Dlx5* within NCCs would result in craniofacial and cardiac NCC phenotypes, we generated a conditional *Dlx5*-gain-of-function mouse line (*CAG-CAT-Dlx5*, Figure 1A). We employed an NCC-specific *Cre* driver (*Wnt1-Cre;* [22]) to ectopically drive *Dlx5* expression within all NCCs (*Wnt1-Cre*;*CAG-CAT-Dlx5* (*Dlx5 NCC oe*). In order to determine if persistent NCC expression of *Dlx5* causes a NCC phenotype, we first looked at craniofacial structures at E16.5 (Figure 1). Compared with *Cre*-negative littermates (Figure 1B), the upper jaw (uj) of *Dlx5 NCC oe* embryos is underdeveloped and split along the midline, which results in a severe midfacial cleft that allows visualization of the tongue (t, Figure 1C). The mandible (md) is also hypoplastic and misshaped (Figure 1D, E). This phenotype is similar to what is observed in HAND1 dimer mutant mice [9] but is far more severe than observed in embryos recently reported in which a *Dlx5* cDNA was inserted into the *ROSA26* locus and then activated in NCCs (*NCC^Dlx5^*) [23].

To determine how these changes were reflected in near term embryos, E18.5 *Dlx5 NCC oe* and *Control* (*Cre*-negative *CAG-CAT-Dlx5* littermates) embryos were stained with Alizarin Red and Alcian Blue to visualize bone and cartilage, respectively. Compared to control embryos (Figure 1C,E), the NCC-derived bones of the skull vault (frontal, f) and nasal, n) and skull base bone (basisphenoid (bs), alisphenoid (al), and palatine (p) bones) are hypoplastic or absent in *Dlx5 NCC oe* embryos (Figure 1D,F). In addition, there is a large midfacial cleft in which the two halves of the premaxilla (pmx) fail to meet at the midline. This cleft extends back through the palatal processes of the maxilla (mx) but does not affect fusion of the palatine bones. The maxilla bones are quite dysmorphic, which makes it impossible to determine if they represent a homeotic transformation into more a mandible-like shape as observed in the *NCC^Dlx5^* mice [23]. However, these changes are similar to those observed when an *Endothelin-1* (*Edn1*) cDNA is driven in NCCs using *Wnt1-Cre* mice [24,25]. In one *Chicken b-actin (CBA)-Edn1;Wnt1-Cre* line, embryos have a classic homeotic transformation of the maxilla into a mandible-like structure while embryos from another line have a large midfacial cleft. Since there is not a significant difference in mature EDN1 by Enzyme-Linked ImmunoSorbent Assay (ELISA) between the two lines, the differences likely reflect insertion site dynamics, with the varying phenotypic outcome reflecting a range of EDN1 action in NCCs. Overall, the phenotype of *Dlx5 NCC oe* embryos indicates that DLX5 has several unrecognized functions during early NCC patterning. A more detailed analysis of this is being reported elsewhere.

### 3.2. NCC Expression of CAG-CAT-Dlx5 Down Regulates Hand1 Ventral Cap Expression

**Figure 2:**
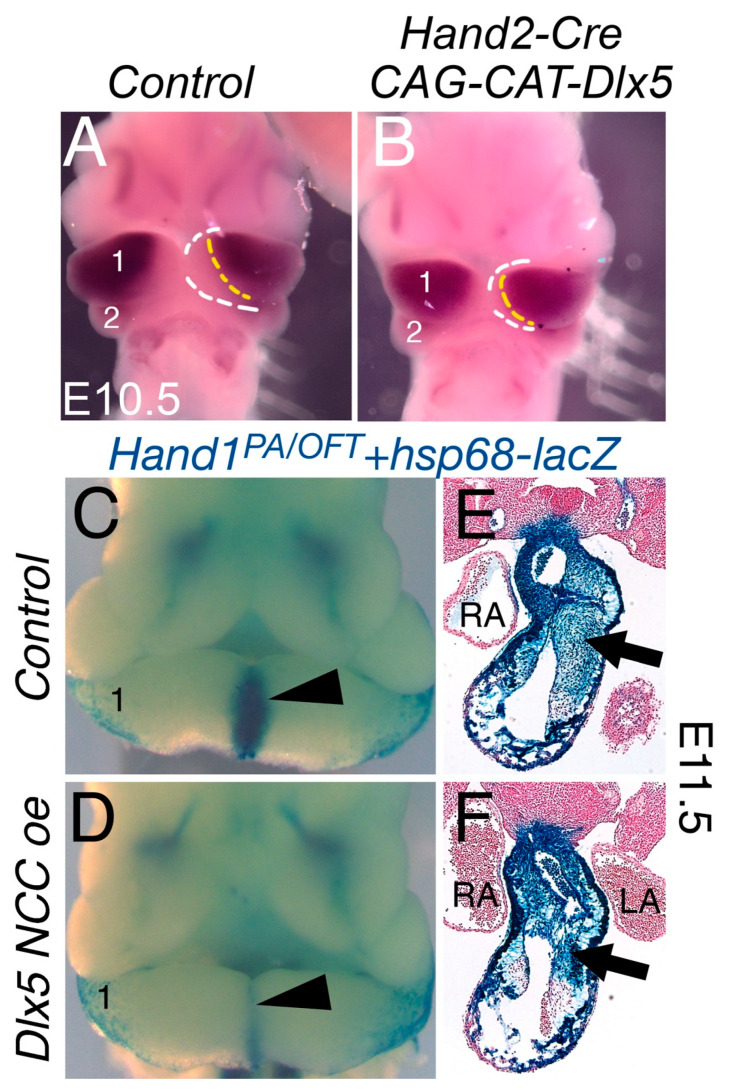
NCC-specific *Dlx5* overexpression represses *Hand1^PA/OFT^* expression. (**A**) E10.5 wholemount in situ hybridization of *Dlx5* in *Control* and *Hand2-Cre; CAG-CAT-Dlx5* embryos. The yellow dotted line marks the ventral most domain of expression and the white dotted line marks the ventral most aspect of the first mandibular arch (1). (**B**) *Hand2-Cre*-activated *Dlx5* transgene expression reveal a measurable ventral extension of *Dlx5* expression into the ventral arch (reduced space between yellow and white lines). (**C**) Wholemount β-galactosidase staining from the *Hand1^PA/OFT^* transgene. The arrowhead marks distal cap expression. (**D**) *Hand1^PA/OFT^*-*lacZ* transgene expression on *Dlx5 NCC oe* (*Wnt1-Cre*; *CAG-CAT-Dlx5*) alleles. Visibly reduced β-galactosidase staining within the distal arch domain is observed. (**E**) Transverse section through the cardiac OFT of *Hand1^PA/OFT^* mice stained for β-galactosidase. Staining is robustly observed within cardiac NCC (arrow) and myocardial cuff. (**F**) *Hand1^PA/OFT^*-*LacZ* transgene expression on *Dlx5 NCC oe* alleles. Staining is similar in intensity to control embryos. However, the ventral extension of β-galactosidase positive NCC is moved dorsally (arrow).

DLX5 regulates gene expression in post-migration cranial NCCs and, although expressed in cardiac NCCs, is not required for OFT morphogenesis, as heart defects are not reported in *Dlx5;Dlx6* double knockout mice [12,23,24,25,26,27]. DLX5 negatively regulates *Hand1* expression [8] and positively regulates *Hand2* expression [6,28] in cranial NCCs. To ensure that the Cre-activated transgene is functional, we first intercrossed *CAG-CAT-Dlx5* mice with the *Hand2-Cre* driver (Figure 2) [3]. E10.5 whole mount in situ hybridization of *Dlx5* shows normal robust dorsal expression where the yellow line demarks its ventral most expression and the white line shows the ventral most cap of the arch of control embryos (Figure 2A). Cre-activation of the *CAG-CAT-Dlx5* allele reveals a noticeable expansion of *Dlx5* expression ventrally such that the space between the yellow and white lined marked boundaries is noticeably reduced, which indicates the efficacy of the Cre-inducible *CAG-CAT-Dlx5* allele (Figure 2B). We next intercrossed *CAG-CAT-Dlx5* mice with our *Hand1^PA/^*^OFT^-*lacZ* reporter line, which we have previously shown is sensitive to DLX5 negative regulation [8]. At E11.5, control embryos show expected cranial and cardiac NCC expression as well as the second heart field derived myocardium of the myocardial cuff (Figure 2C). Most notably, the ventral most NCC of the mandibular arch strongly expresses the *Hand1^PA/^*^OFT^-*lacZ* reporter (1, arrowhead, Figure 2C, n = 4). When *Hand1^PA/^*^OFT^-*lacZ* reporter expression is combined with the *Wnt1-Cre*; *CAG-CAT-Dlx5* alleles, significantly reduced β-galactosidase staining is observed within the ventral cap domain of arch 1 (Figure 2D arrowhead, n = 4). Transverse sections through the cardiac OFT reveal robust β-galactosidase staining within the NCC mesenchyme and OFT myocardium (Figure 2E arrow). In *Dlx5 NCC oe*; *Hand1^PA/OFT^* -*lacZ* mice, the β-galactosidase staining intensity is unchanged. However, the ventral boundary of the β-galactosidase positive NCCs is more dorsal (Figure 2F arrow).

### 3.3. Dlx5 NCC oe OFTs Present Persistent Truncus Arteriosus (PTA)

**Figure 3:**
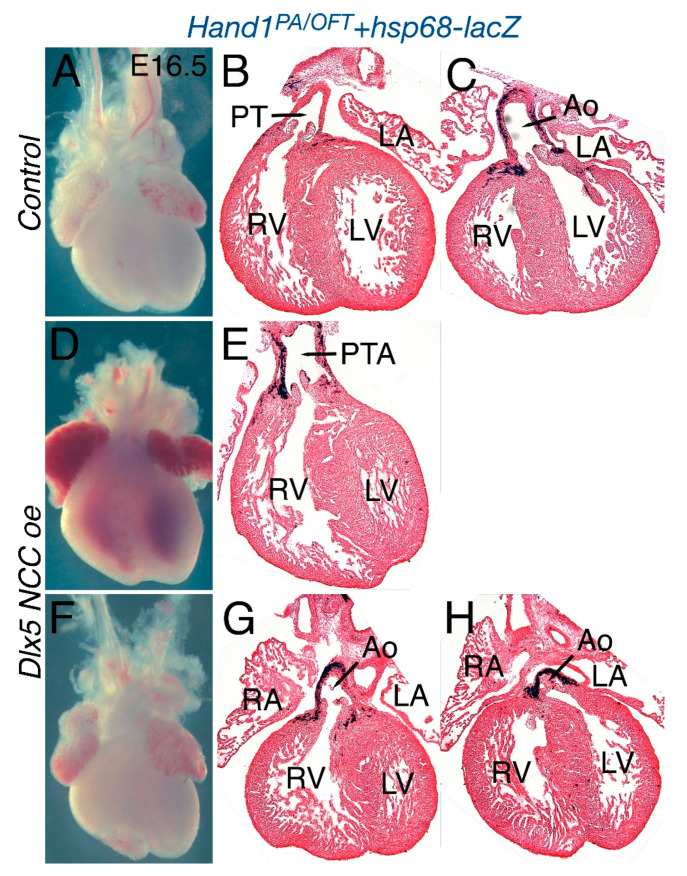
Phenotypic analysis of *Dlx5 NCC oe* hearts at E16.5. Whole mount (**A**) and frontal sections (**B**,**C**) of control (*CAG-CAT-Dlx5*;*Hand1^PA/OFT^*-*lacZ*) heart showing patent septation and proper alignment of the pulmonary trunk (PT) and aorta (Ao) with the right (RV) and left (LV) ventricles. Wholemount *Dlx5 NCC oe* hearts at E16.5 reveal persistent truncus arteriosus (PTA, **D**,**E**) or double outlet right ventricle (**F**–**H**) wherein the aorta is connected to both RV: right ventricle and LV; left ventricle, RA: right atria, LA: left atria.

Disruption of *Hand* factor expression causes cardiac defects within the OFT [4,28]. To determine whether persistent *Dlx5* expression within cardiac NCCs alters OFT morphogenesis and to assay *Hand1* OFT expression, we looked at E16.5 hearts (Figure 3). In control (*CAG-CAT-Dlx5*; *Hand1^PA/OFT^*-*lacZ*) E16.5 hearts, OFT formation appears normal, wherein the pulmonary artery (PT) directly connects with the right ventricle (RV) and the aorta (Ao) directly connects with the left ventricle (LV, Figure 3A–C). *Hand1^PA/OFT^*-*lacZ* expression, visualized by β-galactosidase staining, is detectable within the smooth muscle wall of the aorta and within myocardial cuff cardiomyocytes. In contrast, *Dlx5 NCC oe* mutants present with either a single OFT vessel, which is a condition termed persistent truncus arteriosus (PTA, Figure 3D and E, n = 6/10), or with a double outlet right ventricle (DORV), wherein the Ao connects directly with both right ventricle (RV) and left ventricle (LV) (Figure 3F–H, n = 3/10). *Hand1^PA/OFT^*-*lacZ* expression is observed within the aortic smooth muscle and cuff myocardium. Summary of the encountered phenotypes is presented in Table 1.

### 3.4. NCC Migration into the OFT Is Limited in Dlx5 NCC oe Mice

**Figure 4:**
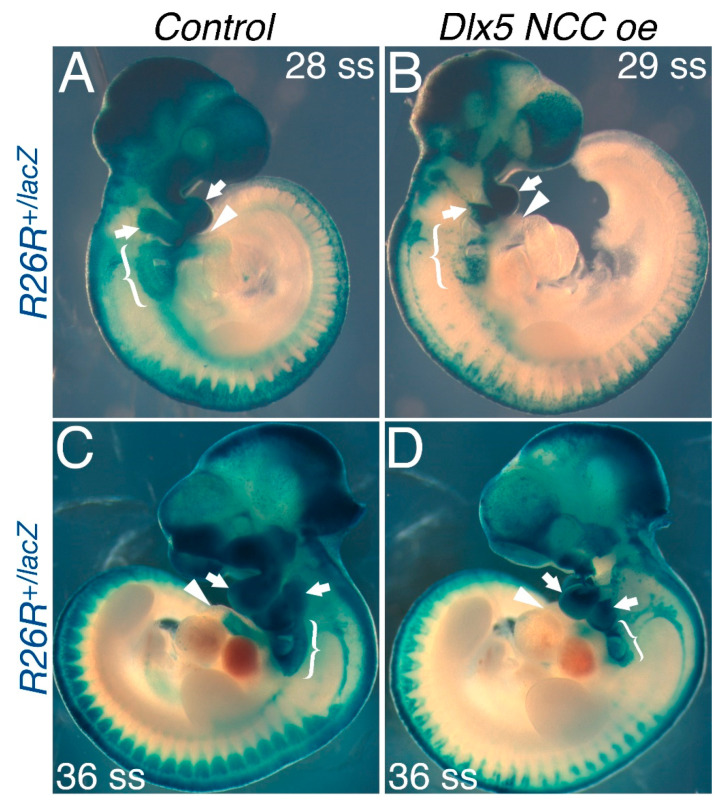
NCC lineage analysis of *Dlx5 NCC oe* E9.5 and E10.5 embryos. (**A**) E9.5 *Wnt1-Cre; R26R^lacZ/+^* embryo stained for β-galactosidase activity. Blue stained cells represent NCC migration through the embryo. Cranial NCCs populating the first and second pharyngeal arches are noted by arrows. Cardiac NCCs occupying the caudal pharyngeal arches are denoted by brackets. NCCs within the outflow tract (OFT) are denoted by arrowheads. (**B**) NCC lineage analysis in *Dlx5 NCC oe* embryos shows reduced first and second pharyngeal arch size (arrows) and reduced cardiac NCC number dorsal to and within the OFT. (**C**) Control and (**D**) *Dlx5 NCC oe* embryo NCC lineage analysis at E10.5 demonstrate that the cranial arches are hypoplastic in *Dlx5 NCC oe* embryos when compared to control embryos (arrows). Reduced numbers of cardiac NCCs (brackets) and NCCs within the OFT (arrowheads) are also observed.

The PTA and DORV observed in *Dlx5 NCC oe* mutants could be due to do altered NCC morphogenesis, increased NCC cell death, or lack of normal NCC migration. To determine what mechanisms are involved, we first performed a *Wnt1-Cre* lineage analysis between E9.5 and E11.5 to look for migration of *Wnt1-Cre* lineage cells within the OFT (Figure 4). In both E9.5 (28 and 29 somite) and E10.5 (36 somite) embryos, both the first and second pharyngeal arches are robustly positive for *Wnt1-Cre* marked NCCs (Figure 4A,C, arrows). Cardiac NCCs are also robustly present dorsal to the OFT (bracket) and can be seen entering the OFT (arrowhead, Figure 4A,C). In *Dlx5 NCC oe* mutants, *Wnt1-Cre* marked NCCs are also observed. However, β-galactosidase staining is less robust (Figure 4B,D). First and second phalangeal arches are smaller (Figure 4B,D, arrows). The population of cardiac NCCs dorsal to the OFT is smaller (Figure 4B,D, bracket) and the *Wnt1-Cre* marked NCCs visible within the OFT are clearly diminished (Figure 4B,D, arrowheads).

### 3.5. Dlx5 NCC oe Embryos Exhibit NCC Cell Death Within the Neural Tube, Pharyngeal Arches but Minimally Within the OFT

**Figure 5:**
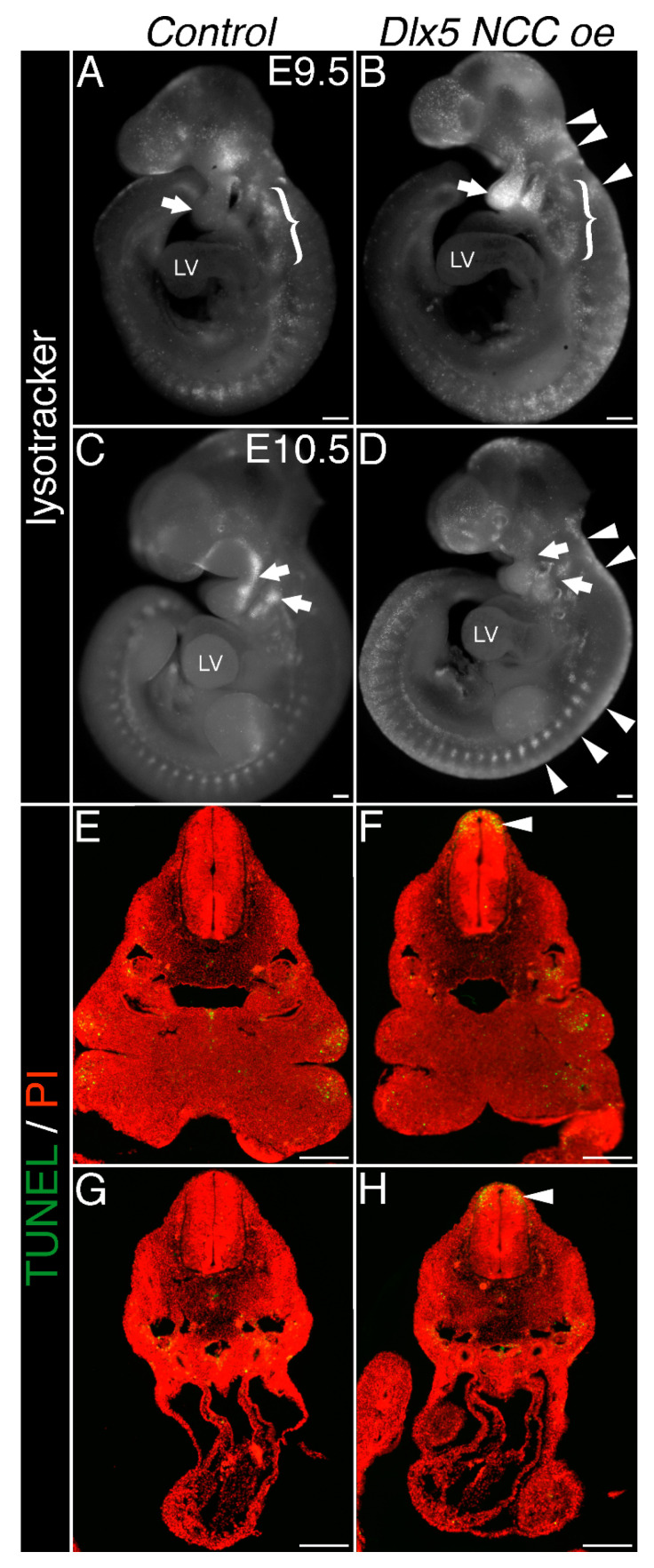
Lysotracker and Terminal deoxynucleotidyl transferase. dUTP nick end labeling (TUNEL) staining of *Dlx5 NCC oe* embryos. (**A**) E.9.5 control embryo showing normal cell death within the head and tissues dorsal to the OFT (brackets). No significant apoptosis is observed within the first and second pharyngeal arches (arrow). (**B**) E9.5 *Dlx5 NCC oe* embryo showing visible cell death within the neural tube (arrowheads) and, most notably, within the first and second pharyngeal arches (arrow). (**C**) E10.5 control embryo showing normal cell death. Arrows denote cell death within the proximal rostral pharyngeal domain of the first and second pharyngeal arches. (**D**) E10.5 *Dlx5 NCC oe* embryo showing persistent dorsal neural tube cell death, whereas first and second pharyngeal arches show less apoptosis than controls. (**E**) and (**F**) E10.5 TUNEL stained control and *Dlx5 NCC oe* embryos. Elevated cell death within the neural tube of *Dlx5 NCC oe* embryos is clearly visible (arrowhead), as are the hypoplastic first and second pharyngeal arches. (**G**,**H**) more caudal sections showing minimal cell death within the cardiac OFT in control and *Dlx5 NCC oe* embryos. Scale bars = 200 μm.

To determine whether *Wnt1-Cre* marked NCC reduction within the pharyngeal arches reflects solely migration defects and not NCC cell death, we performed lysotracker staining to assess cell death in E9.5 and E10.5 *Dlx5 NCC oe* embryos. In E9.5 controls, developmentally normal cell death is observed with regions of the head as well as tissues located dorsally to the OFT (Figure 5A, bracket). Cell death within the pharyngeal arches is minimal (arrow Figure 5A, n = 4). In contrast, significant cell death is observed at the dorsal neural tube (arrowheads) and within the first and second pharyngeal arches (arrow robust positive staining within the arches, Figure 5B, n = 4). Cell death in the caudal pharyngeal arches was not appreciably affected in E9.5 *Dlx5 NCC oe* embryos (Figure 5A,B, brackets, n = 4). No significant cell death is observed within the heart. At E10.5, control (*CAG-CAT-Dlx5*) embryos displayed domains of lysotracker-positive cells within the proximal rostral pharyngeal arches (Figure 5C, white arrows). Although the pharyngeal arches are now hypoplastic at this stage, the lysotracker-positive cells of proximal rostral pharyngeal arches of *Dlx5 NCC oe* littermates are not observed (Figure 5D, white arrows). Continued cell death along the dorsal neural tube is still evident (arrowheads, Figure 5D). To characterize this aberrant cell death in closer detail, we performed TUNEL analyses upon embryonic sections at E10.5. (Figure 5E–H). Compared to control embryos, extensive cell death in the dorsal neural tube is visible in the *Dlx5 NCC oe* embryos at E10.5 (compare Figure 5E,F, arrowheads, n = 5). The cranial pharyngeal arches are also clearly hypoplastic in E10.5 *Dlx5 NCC oe* embryos. No significant changes in cell death are observed in E10.5 OFT of *Dlx5 NCC oe* embryos when compared to control embryos. Together, this data shows that decreased NCC contribution to the pharyngeal arches and OFTs of *Dlx5 NCC oe* embryos is mechanistically likely the result of the increased NCC death observed during NCC migration that initiates at their point of origin within the dorsal neural tube.

**Figure 6:**
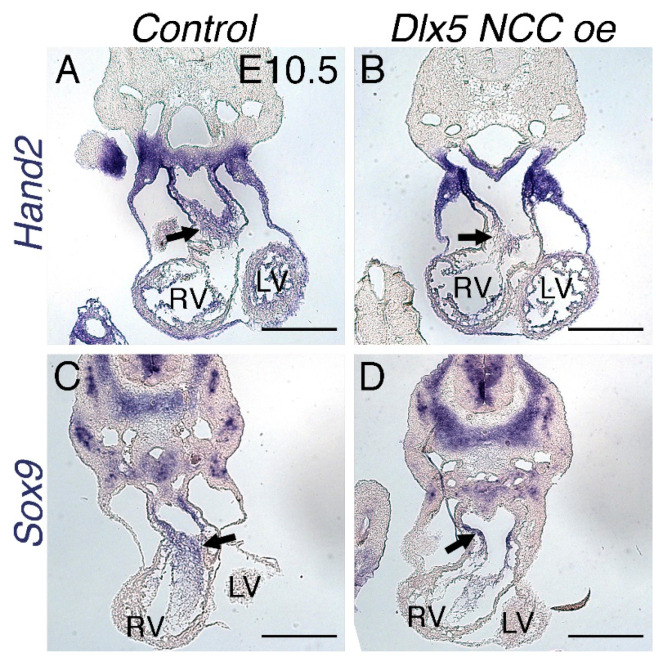
Expression of the OFT markers *Hand2* and *Sox9* is reduced within *Dlx5 NCC oe* embryos. (**A,B**) In situ hybridization showing expression of *Hand2* within control (**A**) and *Dlx5 NCC oe* (**B**) E10.5 embryo hearts. (**C**,**D**) E10.5 control (**C**) and *Dlx5 NCC oe* (**D**) embryos showing *Sox9* mRNA expression. Arrows denote cardiac NCC. Scale bars = 250 μm.

To determine whether gene expression is altered in the *Dlx5 NCC oe* NCCs that contribute to the OFT, we looked at the expression of the cardiac NCC and OFT myocardium marker *Hand2* [28,29] and the NCC marker *Sox9* [30,31] (Figure 6). Expression of *Hand2* is modest within the ventricular myocardium of the RV and LV and more robust within the endocardium, the myocardial cuff myocardium, and cardiac NCC (arrow) of control E10.5 hearts (Figure 6A). In *Dlx5 NCC oe* hearts, expression within the endocardium, ventricles, and myocardial cuff is similar to that of control hearts. However, expression within the cardiac NCC is diminished (arrow, Figure 6B). Robust *Sox9* expression is observed within control OFT NCCs (arrow, Figure 6C), whereas *Sox9* expression with *Dlx5 NCC oe* OFT NCCs is significantly reduced, which results from decreased expression and / or less *Sox9*-expressing NCCs (arrow, Figure 6D).

### 3.6. Persistent Dlx5 Expression Does Not Induce NCCs to Adopt a Neuronal Cell Fate

**Figure 7:**
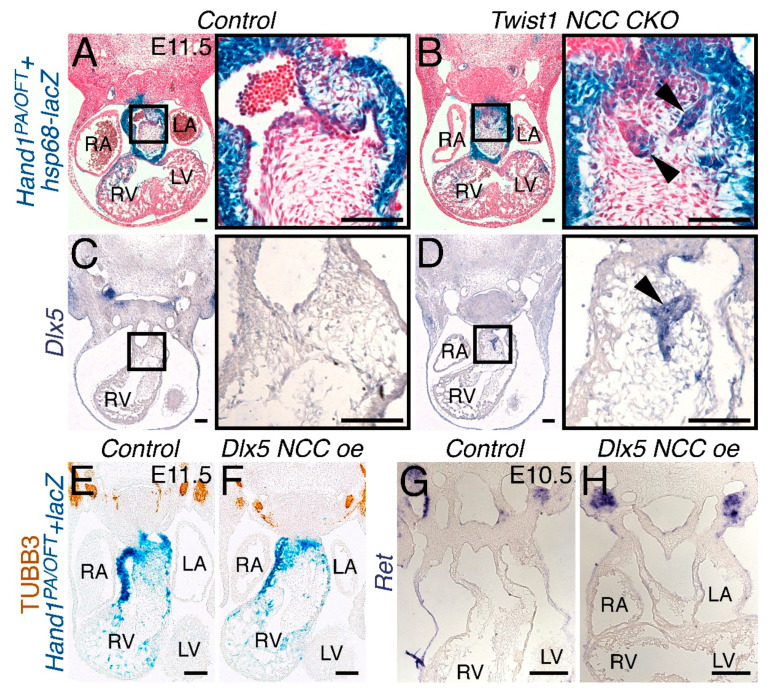
Ectopic *Dlx5* expression within the NCC is not sufficient to induce neurogenesis. (**A, B**) E11.5 *Hand1^PA/OFT^*-*lacZ* stained control (**A**) and *Wnt1-Cre(+); Twist1*^–*/fx*^ mutant (**B**) embryos showing transgene-positive ectopic ganglia within the cardiac NCCs occupying the OFT. (**C**, **D**) In situ hybridization to detect *Dlx5* mRNA shows no expression in control OFTs (**C**) but does show robust expression within the trans-differentiated cardiac NCCs of *Twist1* conditional knockouts (**D**). Immunohistochemistry of pan-neuronal TUBB3 in both control (**E**) and *Dlx5 NCC oe* (**F**) sections reveals no ectopic neuronal staining in the E11.5 OFT. In situ hybridization to detect *Ret* mRNA similarly reveals no neuronal cells within the OFTs of E10.5 control (**G**) or *Dlx5 NCC oe* (**H**) embryos. Scale bars = 100 µm.

Loss of TWIST1 function induces NCCs to differentiate along a neuronal cell fate [13]. Within the OFT, *Twist1*-null cardiac NCCs organize into ganglia-like structures and express a number of neuronal genes [13]. Additionally, these *trans*-differentiating cardiac NCCs express *Hand1* [13]. Crossing the *Hand1^PA/OFT^*-*lacZ* reporter onto a *Twist1*^–*/fx*^, *Wnt1-Cre(+)* background (*Twist1 NCC CKO*) reveals that these NCCs are specifically marked by *Hand1^PA/OFT^* enhancer activity (Figure 7B, arrowheads). Intriguingly, these ganglia-like structures also express *Dlx5* (Figure 7D, arrowhead, n = 3), which is not detectable in the cardiac NCCs of control OFTs (Figure 7C). To determine whether persistent *Dlx5* expression within the NCCs promotes cardiac NCC populations to differentiate along a neuronal path, we looked at the expression of the pan-neuronal marker Class III β-TUBULIN (TUBB3) [32] and the receptor tyrosine kinase *Ret*, which marks NCC-derived neurons [33]. TUBB3 immunostaining of E11.5 embryos on the *Hand1^PA/OFT^*-*lacZ* reporter background revealed no detectable TUBB3 protein within OFT NCCs of either control or *Dlx5 NCC oe* embryos (Figure 7E,F, n = 2). Ret in situ hybridization of E10.5 embryos yielded similar findings (Figure 7G,H, n = 4). These results show that, although *Dlx5* expression is induced in ectopic neurons within *Twist1*-null cardiac NCCs, ectopic *Dlx5* expression within the cardiac NCCs is not sufficient to induce neurogenesis.

### 3.7. Persistent Dlx5 Expression within NCCs Downregulates Dlx6 Expression

**Figure 8:**
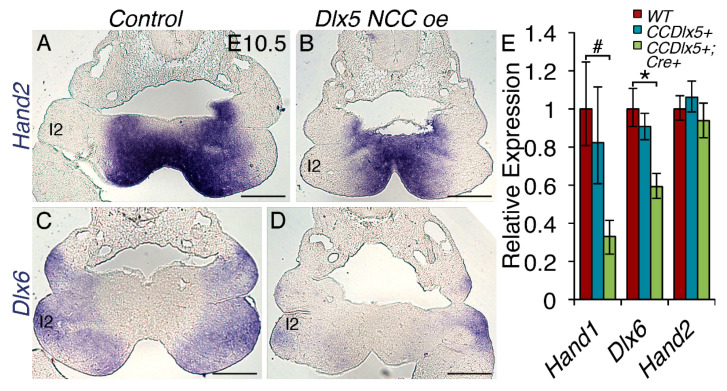
*Dlx5* NCC overexpression downregulates *Dlx6* within the first pharyngeal arch. (**A**, **B**) *Hand2* in situ hybridization showing normal first arch expression in both control (**A**) and *Dlx5 NCC oe* E10.5 embryos. *Dlx6* in situ hybridization showing altered first arch expression when comparing control (**C**) and *Dlx5 NCC oe* embryos (**D**). (**E**) qRT-PCR analyses of *Hand1*, *Dlx6*, and *Hand2* expression from E10.5 first pharyngeal arches. Significant decreases in *Hand1 (# p* ≤ 0.01) and *Dlx6* (* *p* ≤ 0.05) are observed. No significant changes in the *Hand2* message are detected. Scale bars = 250 μm.

Signaling from the Endothelin receptor type A (EDNRA) induces *Dlx5* expression in the pharyngeal arches [34]. In order to see whether DLX5 overexpression had regulatory effects upon other Endothelin 1-induced genes, we looked at expression of *Hand2* and the related homeobox transcription factor *Dlx6* in the pharyngeal arches of the E10.5 control and *Dlx5 NCC oe* embryos (Figure 8). In situ hybridization of *Hand2* mRNA revealed that *Hand2* expression appears unchanged within the first pharyngeal arch (1) in *Dlx5 NCC oe* embryos (Figure 8A,B, n = 3). This is confirmed quantitatively by qRT-PCR (Figure 8E, n = 6). In contrast, *Dlx6* expression is robust within the first pharyngeal arch of control embryos but is significantly reduced in in *Dlx5 NCC oe* embryos (Figure 8C,D,E). *Hand1*, which is known to be negatively regulated by DLX5 [8], is also significantly downregulated (Figure 8E).

## 4. Discussion

NCCs are a dynamic and multipotent cell population that, during embryogenesis, migrate ventrally from the neural tube to contribute to organ morphogenesis [35]. Major components of the cardiac OFT and nearly the entire vertebrate facial complex are NCC-derived. Dysregulation of these NCC populations result in the majority of congenital abnormalities encountered in humans. In this study, we set out to interrogate how NCC gene regulatory networks that include HAND transcription factors facilitate NCC specialization into specific tissue fates. *Hand1* and *Hand2* mark both cranial and cardiac NCC populations [36,37], exhibit genetic interactions that, when disrupted, result in a phenotype [4,19,21,38], and set up tissue boundaries that are essential normal tissue morphogenesis within the post migration NCCs occupying the pharyngeal arches [6,8].

The cranial transcriptional enhancers that drive *Hand1* and *Hand2* within NCC as well as the cardiac NCC transcriptional enhancer for *Hand1* are established [2,5,39]. Analysis of these enhancers has revealed transcriptional inputs from both Endothelin Receptor A EDNRA (through DLX5 and DLX6) and BMP signaling (through SMADs 1/5/8) as well as direct and required regulation of *Hand1* by HAND2 and DLX5/6 [8,10,39]. Given the spatial changes in *Hand1* expression that result from BMP gain-of-function and HAND2 loss-of-function, ectopic expression of DLX5 within all NCC was performed to look at the effects on cranio-facial and OFT formation.

The first observation that is noted is that *Dlx5 NCC oe* mice exhibit severe midface clefting (Figure 1). We speculate that mechanistically this is likely the result of increased cranial NCC apoptosis (Figure 4). *Hand1* phospho mutant conditional knock-in mice exhibit a similar craniofacial phenotype [9]. Although *Hand1* NCC loss-of-function mice exhibit no observable phenotypes [4], it is clear that *Hand1* is down regulated within *Dlx5 NCC oe* mice (Figure 2 and Figure 8). If other potential HAND1 bHLH partners are also transcriptionally regulated, the combination of these changes to the bHLH gene regulatory networks via alteration of the bHLH dimer pool available to form transcriptional dimer complexes could account for the similar phenotypes. Of note, a similar study employing a *Rosa* locus *Dlx5* knockin was recently reported [12,23,24,25,26] and showed short snout, open eyelids, misaligned vibrissae, and a cleft palate with no clear signs of palatine rugae. In our study, using a traditional transgenic insertion increases severity and is likely the result of increased *Dlx5* expression in our model.

The *Hand1^PA/OFT^* enhancer drives expression in both cranial and cardiac NCCs, and DLX5 and DLX6 directly repress *Hand1^PA/OFT^* enhancer activity [8]. However, *Dlx5* and *Dlx6* are not robustly expressed within cardiac NCC populations and *Dlx5/Dlx6* loss-of-function or gain-of-function mutants display no observable cardiac phenotypes [12,23,24,25,26]. Moreover, cardiac NCC expression of *Dlx5* was observed in chicken [12,23,24,25,26] where differences between mammals were observed [35]. When DLX5 is expressed with cardiac NCC, although significant OFT abnormalities like PTA and DORV are observed, the expression of the *Hand1^PA/OFT^* enhancer is not clearly downregulated. This suggests that, although DLX5 is a dominate repressor in cranial NCC populations, its presence in cardiac NCCs does not impact *Hand1^PA/OFT^* enhancer activity, which suggests that DLX5 may not be sufficient for *Hand1* transcriptional repression and that additional factors are required. Along these lines, it is important to note that HAND2 is necessary for *Hand1* expression within cranial but not cardiac NCC. This also suggests that HAND2 must act with additional factors to regulate *Hand1* within the cardiac NCCs. GATA transcription factors are also required for *Hand1^PA/OFT^* transcriptional activity [8]. In E10.5 reporter embryos in which GATA *cis*-regulatory elements in the *Hand1^PA/OFT^* enhancer have been mutagenized, cranial *Hand1^PA/OFT^* expression is ablated whereas cardiac NCC expression, although slightly reduced, persists [8]. Thus, factors required for enhancer activation in one NCC population (DLX5, SMAD, HAND2, GATA) do not significantly alter expression within another NCC population. Cranial NCC subpopulations share prepatterned chromatin states that are poised to respond to distinct local signaling cues depending upon where in the head they ultimately reside [40]. We propose that distinctions between cranial and cardiac NCC chromatin states enable these populations to respond to identical transcriptional inputs within the same cis-regulatory element in unique manners.

Given that there are observed OFT phenotypes, it is clear that ectopic DLX5 activity alters cardiac NCC gene expression. There is clear reduction in *Hand2* expression within cardiac NCCs at E10.5 as well as a reduction of *Sox9* expressing cells (Figure 6). Changes in the cardiac NCC gene regulatory network combined with reductions in NCC numbers are the likely causes of the PTA and DORV observed in *Dlx5 NCC oe* embryos. To assess potential NCC *trans*-differentiation, we looked at the neuronal markers TUBB3 and *Ret*, and found that, even though *Dlx5* expression is highly upregulated in *Twist1*-null cardiac NCCs, upregulation of DLX5 alone is insufficient to cause trans-differentiation (Figure 7).

Lastly, it is clear that *Dlx* gene dosage is modulated in *Dlx5 NCC oe* mice. The highly related *Dlx6*, which is co-expressed with *Dlx5* in cranial NCC, is significantly downregulated within the first and second pharyngeal arches of *Dlx5 NCC oe* embryos (Figure 8). A precise balance of specific transcription factors within subpopulations of NCCs appears necessary for these cells to migrate and differentiate to the correct tissue type and structures. NCC specification is thought to be governed from the rostral-caudal origin of delaminating NCCs from the neural tube. However, post migratory trans-differentiation is possible [13], which reflects the necessity for both positional and gene expression modulation. The data from this study reflects that altering the gene regulatory networks by transcription factor gain-of-function analysis can be used to reveal sensitive and insensitive actions of a single factor on a single enhancer in two separately fated populations of NCCs.

## Figures and Tables

**Table 1:** Outflow tract (OFT) defects observed in *Dlx5* neural crest cell (NCC) oe mutants.

Genotype	n	PTA + VSD	DORV + VSD	Phenotypically Normal
*Wnt1-Cre*	1	0 (0%)	0 (0%)	1 (100%)
*CAG-CAT-Dlx5*	3	0 (0%)	0 (0%)	3 (100%)
*CAG-CAT-Dlx5*; *Wnt1-Cre*	10	6 (60%)	3 (30%)	1 (10%)

DORV, double outlet right ventricle, PTA, persistent truncus arteriosus, and VSD, ventricular septal defect.
